# The Use of Kernel Density Estimation to Examine Associations between Neighborhood Destination Intensity and Walking and Physical Activity

**DOI:** 10.1371/journal.pone.0137402

**Published:** 2015-09-10

**Authors:** Tania L. King, Lukar E. Thornton, Rebecca J. Bentley, Anne M. Kavanagh

**Affiliations:** 1 Gender and Women’s Health, Centre for Health Equity, Melbourne School of Population Health, University of Melbourne, Melbourne, Victoria, Australia; 2 Centre for Physical Activity and Nutrition Research, School of Exercise and Nutrition Sciences, Deakin University, Burwood, Victoria, Australia; Indiana University, UNITED STATES

## Abstract

**Background:**

Local destinations have previously been shown to be associated with higher levels of both physical activity and walking, but little is known about how the distribution of destinations is related to activity. Kernel density estimation is a spatial analysis technique that accounts for the location of features relative to each other. Using kernel density estimation, this study sought to investigate whether individuals who live near destinations (shops and service facilities) that are more intensely distributed rather than dispersed: 1) have higher odds of being sufficiently active; 2) engage in more frequent walking for transport and recreation.

**Methods:**

The sample consisted of 2349 residents of 50 urban areas in metropolitan Melbourne, Australia. Destinations within these areas were geocoded and kernel density estimates of destination intensity were created using kernels of 400m (meters), 800m and 1200m. Using multilevel logistic regression, the association between destination intensity (classified in quintiles Q1(least)—Q5(most)) and likelihood of: 1) being sufficiently active (compared to insufficiently active); 2) walking≥4/week (at least 4 times per week, compared to walking less), was estimated in models that were adjusted for potential confounders.

**Results:**

For all kernel distances, there was a significantly greater likelihood of walking≥4/week, among respondents living in areas of greatest destinations intensity compared to areas with least destination intensity: 400m (Q4 OR 1.41 95%CI 1.02–1.96; Q5 OR 1.49 95%CI 1.06–2.09), 800m (Q4 OR 1.55, 95%CI 1.09–2.21; Q5, OR 1.71, 95%CI 1.18–2.48) and 1200m (Q4, OR 1.7, 95%CI 1.18–2.45; Q5, OR 1.86 95%CI 1.28–2.71). There was also evidence of associations between destination intensity and sufficient physical activity, however these associations were markedly attenuated when walking was included in the models.

**Conclusions:**

This study, conducted within urban Melbourne, found that those who lived in areas of greater destination intensity walked more frequently, and showed higher odds of being sufficiently physically active–an effect that was largely explained by levels of walking. The results suggest that increasing the intensity of destinations in areas where they are more dispersed; and or planning neighborhoods with greater destination intensity, may increase residents’ likelihood of being sufficiently active for health.

## Introduction

Physical inactivity is one of the key lifestyle and societal factors associated with many non-communicable diseases such as obesity, cardiovascular disease, diabetes and metabolic syndrome, that continue to rise in high income countries, and increasingly in the low-middle income countries [1–3]. Walking is the most common form of physical activity in many countries [4–6], and is likely to make a substantial contribution to overall physical activity levels. There is evidence that the majority of physical activity takes places in the neighborhood environment [7, 8] and that characteristics of the built environment are associated with walking [9, 10] and overall physical activity [11–13].

Unlike immutable individual characteristics such as age and sex, many aspects of the built environment are modifiable and therefore amenable to intervention. Local places to walk to such as shops, services and transport stops (hereafter referred to as destinations) have been shown to be associated with the frequency and time spent walking [14, 15] when measured in terms of the presence or number within a defined area. It is possible through urban planning to design where, and how many, destinations are in areas.

Destinations are distributed throughout neighborhoods in a myriad of different ways, and little is known about how their distribution (i.e. mixed types, dispersed vs. clustered) might differentially influence walking in local areas. A small number of studies have looked beyond the presence or number of destinations, and have created composite measures of destination distribution that combine mix, presence and number [16, 17]. The dearth of research examining the ways that destination distribution affects walking and physical activity has been recognized [16], with calls for more research in the area [18].

There are other limitations and deficits in the literature in relation to the association between destinations and physical activity. Access to destinations in neighborhoods has typically been measured in terms of the destinations present within a defined catchment or buffer (i.e. a count of the number of destinations within a certain distance of home). This approach has been criticized because a feature (in this case destination) is simply classified as present or absent [19, 20]. The binary nature of such access measures may obfuscate or ignore the more graded shift from what is accessible, to what is not [21]. A destination or activity located at the edge of the areal unit is not equivalent to a destination located at its center, however typical binary measures do not accommodate this, and analyze them as if their effect is the same. Another major criticism is that such measures of accessibility do not take into account the location of destinations relative to each other (i.e. they provide no indication of whether they are intensely distributed or dispersed).

Kernel density estimation (KDE) is a spatial method that accounts for the location of features (i.e. destinations) relative to each other, and offers a more graded measure of destination accessibility. It has been used to examine attributes of the environment such as health resources [22], the food environment [23–25] and park access [20]. It improves on count or proximity measures of access by transforming point data onto a continuous surface [26], thereby enabling the density of a feature to be estimated at any point on the map surface [19]. While KDE has been used to investigate the distribution of destinations such as food stores [27–29], in relation to outcomes such as BMI [29], obesity [28], and dietary intake [27], we are only aware of one study that has applied KDE (of recreational resources) in relation to a physical activity outcome [30].

In this study we investigated associations between the distribution of destinations and two outcomes, walking frequency and physical activity sufficiency, using KDE with three different kernel sizes (400m, 800m and 1200m). Applied to the distribution of destinations, KDE provides an estimate of the proximity and density of destinations in relation to respondent houses—we refer to this as destination intensity. The study addresses the following research questions:

1. Is destination intensity associated with overall physical activity?
2. Knowing that walking is the most common form of physical activity, is destination intensity associated with increased levels of walking?
3. Does level of walking explain the associations between destination intensity and physical activity?

## Methods

The analyses are based on individual and area-level data collected as part of the Victorian Lifestyle and Neighborhood Environment Study (VicLANES) from 2349 individuals in 50 small areas in metropolitan Melbourne. Additional information on areas was also obtained from a range of different administrative geospatial datasets.

### Study design

VicLANES was a large, multilevel study that was conducted in 2003–2004 across the 21 innermost local government areas (LGAs) in Melbourne, Australia. The VicLANES methods have been reported previously [31, 32]. Briefly, census collection districts (known as CCDs, at the time of the study these were the smallest geographic unit of measurement used by the Australian Bureau of Statistics (ABS)) were ranked according to a household measure of low income (<$400/week), then stratified into septiles. Fifty CCDs were then randomly selected from the top (17), middle (16) and bottom (17) septile. Postal surveys were sent to 4005 residents over the age of 18 years, who were randomly selected from the electoral roll (voting is compulsory for all Australians over the age of 18 years, and it is estimated that 97.7% of those eligible to vote are enrolled do so) [33]. The Tailored Design Method for Mail Surveys [34] was adopted to maximize response rates. A 58.7% valid completion rate was achieved, with 2349 residents returning a valid survey about their physical activity behavior. Participation rates were inversely associated with area disadvantage, with higher response rates observed in the most advantaged areas; and the most disadvantaged areas having the lowest response rates [32].

### Ethics Statement

The VicLANES project design was approved by the La Trobe University Human Ethics Committee (#02–130). Participants received an information pack, along with their survey in the mail. This advised them: of the risks and benefits of participating; that their participation was voluntary; of the ways that the data would be used; of the strict procedures to protect confidentiality and ensure anonymity. Return of completed surveys was considered indicative of consent—a procedure approved by the Latrobe University Human Ethics Committee.

### Outcome measures

#### Walking

A closed response question asked respondents about their frequency of walking for ≥10 minutes in the previous month. Respondents were required to tick one of six response categories: never, about once or twice, about once a week, about 2–3 times a week, about 4–5 times a week, every day. Responses to this question about walking frequency were dichotomized to ‘three times a week or less’ (≤3/week) and ‘four times a week or more’ (≥4/week). Using this dichotomization, the cut-off response category for the greater walking category (“4–5 times a week”), closely approximates the number of sessions (at least five) recommended to meet physical activity sufficiency [5, 35].

#### Physical Activity Sufficiency

Using items from the Active Australia Survey, respondents were asked to indicate the frequency and duration of their participation in walking, vigorous physical activity, moderate physical activity vigorous garden or yard work. These items were then used to produce a measure of overall physical activity sufficiency. The Active Australia Questionnaire has been used in national surveys, and demonstrates very good reliability and validity[35].

Australian and international guidelines recommend that a person needs to participate in at least 30 minutes of moderate to vigorous intensity activity most days of the week, for a total of at least 150 minutes of activity [5, 35, 36]. According to the Active Australia Survey guidelines, physical activity sufficiency for health can be measured in two ways [35]: 1) measured as total time engaged in physical activity (at least 150 minutes for sufficiency); 2) measured as total time across total number of sessions (at least 150 minutes across at least five sessions). We have chosen to use the combined measure of time and number of sessions (at least 150 minutes of at least moderate intensity activity across at least five session week) [37, 38], because it matches guidelines for physical activity sufficiency.

In accordance with the Active Australia Survey administration and implementation guidelines, VicLANES responses were converted to total amount of time (minutes) engaged in each activity, and summed, with vigorous activity weighted by a factor of two [35, 39]. Respondents were then categorized in one of two categories: those reporting less than 150 minutes of at least moderate activity were classified as insufficiently active; those with at least 150 minutes of at least moderate activity across at least five sessions were classified as sufficiently active.

### Exposure variable: Destinations

Destination information came from two principal sources: 1) the VicLANES environmental audit [40, 41], and 2) publicly available spatial datasets. Destinations included in the analysis were: educational facilities (schools, kindergartens, universities), café/takeaway stores, transport stops and stations, supermarkets, sports facilities, community resources (such as libraries, places of worship, community centers), small food stores (such as convenience stores, bakeries, butchers, green grocers). For a list of destination types and the sources of this destination data refer to S1 Table.

#### Kernel density estimation: Constructing the exposure variable in ArcGIS

In ArcGIS 10.1 [42] all destinations were combined and merged into a single layer. The kernel density surface of destinations was estimated, and extracted using the “extract values to points” command in the Spatial Analyst toolbox in ArcGIS [42].

The process of kernel density estimation commences with a continuous map surface divided into a grid of specified cell sizes. Across this continuous map, KDE fits a series of cones or kernels centered over each feature of interest (in this case destinations), creating a continuous map of feature density or intensity [43]. The radius of each cone/kernel is set to a distance that is estimated to reflect the service area/area of effect of that particular feature or resource. Each cell on the map surface is assigned a kernel density estimate that is weighted according to its proximity to the center of the cone/kernel. Cells at the center of the cone are therefore assigned greater weight, and receive higher estimates; cells at the cone’s periphery are assigned a negligible weighting and receive small estimates [26, 43]. In effect, kernel density estimates are inversely related to the distance from the cone’s center [43]. The cones of different features/destinations overlap, often substantially. A smoothing function (with a bivariate Gaussian distribution) adds the estimates of overlapping kernels for each cell [43, 44].

In this analysis, individual output cells were partitioned at 20m (meters) x20m. Twenty meters provides greater precision in estimates for individuals–partitioning at larger cell sizes would mean that neighboring respondents would get the same estimate.

**Fig 1:**
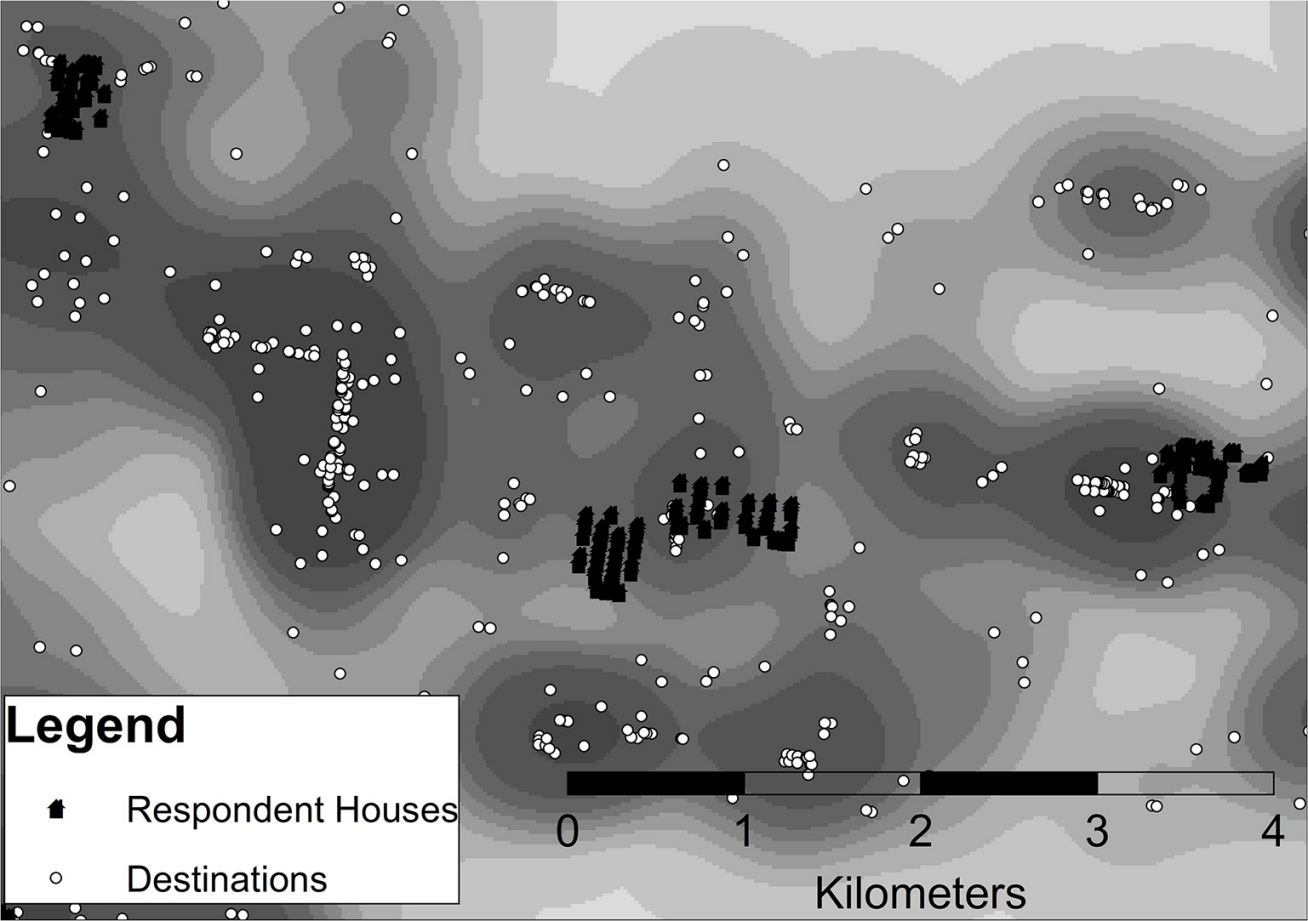
An example of a raster representation of destination distribution using 1200m kernels.

An example of the kernel density output using 1200m kernels is presented below (Fig 1). It shows the distribution of destinations across a continuous surface: areas of greatest destination intensity are represented by the darkest shading, while those areas of few or no destinations are shown in white.

To produce the image above (Fig 1), a kernel with a cone radius of 1200m was placed over every destination in the dataset. Overlapping cones were added to produce a continuous surface, with destinations closest together producing greatest kernel density estimates. Also located on this map space are respondent houses. It is important to note that the kernel density estimates were calculated independent of respondent houses. The kernel density values were extracted so that each respondent’s household location was assigned the kernel density value of the output cell in which they resided. While the estimates are calculated on the basis of destination proximity to each other, the values extracted at each respondent location provide an indication of the proximity and density of destinations in relation to the respondent location. High kernel density estimates indicate high intensity/clustering of destinations that minimize the distance between a respondent’s home and destinations. Low kernel density estimates indicate negligible, dispersed destinations. Moderate kernel density estimates may indicate dispersed destinations, or they may result when a respondent is located some distance from a set of highly clustered destinations.

In this analysis, kernel density estimates were calculated using kernel sizes of 400m, 800m and 1200m.

#### Constructing the exposure variable for statistical analysis

Kernel density estimates were categorized into quintiles as this categorization accommodated the gradations of kernel density estimates and meant that there was no need to make assumptions regarding the association between kernel density and the outcome variables (i.e. linearity). Furthermore, there is a precedent for the categorization of KDE output into quintiles [24].

### Confounders

Based on the literature, several covariates were included in the models as potential confounders because they are likely to be related to physical activity/walking frequency, and destination distribution. These were: age (grouped into six categories: 18–24 years; 25–34 years; 35–44 years; 45–54 years; 55–64 years; 64 years and over), sex, country of birth (born in Australia; born in a country other than Australia), education (bachelor degree or higher; diploma; vocational training; and no post school qualification), household type (single adult-no children; single adult with children; two or more adults-no children; two or more adults with children), dominant household occupation (professional; white-collar employee; blue-collar employee; not in labor force–including retirees, students, unemployed, those not looking for, or unable to work), and disability/injury that prevents exercise (yes, no). Area disadvantage was also included as a potential confounder. The three area septiles used to set the sample frame (see ‘Study design’ above) were used as an indicator of area disadvantage, and were defined as least disadvantaged, mid disadvantaged and most disadvantaged.

### Statistical analysis

Analysis was conducted in 2012–2013. Pregnant women (n = 22) were excluded because their activity levels may have been altered by their pregnancy status. One CCD from just outside the central business district (CBD) of Melbourne was omitted from the final analysis (n = 14) as this CCD’s catchment area encapsulated almost the entire CBD, and the number of features and destinations contained in the catchment area of this CCD was irregularly high. There was no missing data for sex, age group or level of area disadvantage. Missing data for the other variables ranged from 0.5% to 2.9%, with the exception of the disability item, for which missing data amounted to 6.1%. Eight respondents for whom there was no walking data were excluded, resulting in an analytical sample of 2305 respondents, and 49 CCDs for the walking analysis. For the physical activity analysis, data was missing from 373 respondents (15.9%), resulting in an analytical sample of 1976.

All analyses were conducted in Stata IC 10.0. The referent category for the walking outcome was the lowest walking category (walking three times a week or less). For the physical activity outcome, insufficient physical activity was the referent category, while the referent category for the exposure was Q1 (quintile 1, lowest destination intensity). Descriptive analyses included cross tabulations between the outcomes and both individual covariates and kernel density estimates. Using the xtmelogit commands in Stata, multilevel logistic regression was performed (with CCDs at level 2 and individuals at level 1) to examine the associations between both the physical activity and walking outcomes, and the three kernel density measures (400m, 800m, and 1200m). All models adjusted for confounders. Mediation analysis was also conducted: we included the walking variable in the physical activity models to see whether walking explained the physical activity estimates. Odds ratios and 95% confidence intervals are reported for all estimates.

## Results

### Descriptive statistics

Sample characteristics by frequency of walking and physical activity sufficiency are presented in Table 1. Physical activity sufficiency showed patterning by the socio-demographic characteristics, with those aged 45–54 years, and over 65 reporting higher levels of physical activity sufficiency. A higher proportion of those: born in Australia, with at least a bachelor degree, for whom the dominant household occupation was professional, living in a household with no children, living in areas of mid or least disadvantage were sufficiently physically active.

**Table 1:** Summary Statistics of Dataset.

		Walking frequency			Physical activity sufficiency		
Variable	Response category	Total	3 times a week or less n(%)	4 times a week or more	Total n(%)	Insufficient level of physical activity	Sufficient level of physical activity
				n(%)		n(%)	n(%)
	TOTAL	2305	1306 (56.7)	999 (43.3)	1976	1088(55.1)	888(44.9)
**Sex**	Male	1015	592(58.3)	423(41.7)	866	476(55.0)	390(45.0)
	Female	1290	714(55.4)	576(44.7)	1110	612(55.1)	498(44.9)
		Pearson chi2(1) = 2.0491 Pr = 0.152			Pearson chi2(1) = 0.0057 Pr = 0.940		
**Age (years)**	18–24	182	113(62.1)	69(37.9)	161	63(39.1)	63(39.1)
	25–34	395	254(64.3)	141(35.7)	363	198(54.6)	165(45.5)
	35–44	492	306(62.2)	186(37.8)	430	261(60.7)	169(39.3)
	45–54	495	268(54.1)	227(45.9)	424	219(51.7)	205(48.4)
	55–64	391	194(49.6)	197(50.4)	334	182(54.5)	152(45.5)
	Over 65	350	171(48.9)	179(51.1)	264	130(49.2)	134(50.8)
		Pearson chi2(5) = 35.5762 Pr = 0.000			Pearson chi2(5) = 13.4043 Pr = 0.020		
**Country of**	Elsewhere	663	385(58.1)	278(41.9)	550	339(61.6)	211(38.4)
**birth**	Australia	1631	914(56.0)	717(44.0)	1417	744(52.5)	673(47.5)
		Pearson chi2(1) = 0.7910 Pr = 0.374			Pearson chi2(1) = 13.3506 Pr = 0.000		
**Education**	Bachelor degree or higher	719	423(58.8)	296(41.2)	632	308(48.7)	324(51.3)
	Diploma	257	148(57.6)	109(42.4)	223	124(55.6)	99(44.4)
	Vocational	431	242(56.2)	189(43.9)	373	212(56.8)	161(43.2)
	No post school qual.	831	455(54.8)	376(45.3)	693	411(59.3)	282(40.7)
		Pearson chi2(3) = 2.7471 Pr = 0.432			Pearson chi2(3) = 15.7514 Pr = 0.0012		
**Dominant**	Professionals	1060	630(59.4)	430(40.6)	942	484(51.4)	458(48.6)
**Occupation**	White-collar	352	188(53.4)	164(46.6)	313	176(56.2)	137(43.8)
**(household)**	Blue-collar	243	153(63.0)	90(37.0)	199	138(69.4)	61(30.7)
	Not in labor force	597	305(51.1)	292(48.9)	483	267(55.3)	216(44.7)
		Pearson chi2(3) = 16.3137 Pr = 0.001			Pearson chi2(3) = 21.7417 Pr = 0.000		
**Household**	Single adult, no children	397	199(50.1)	198(49.9)	326	165(50.6)	161(49.4)
**type**	Single adult, children	133	88(66.2)	45(33.8)	116	76(65.5)	40(34.5)
	2+ adults, no children	947	517(54.6)	430(45.4)	810	421(52.0)	389(48.0)
	2+ adults, children	778	473(60.8)	305(39.2)	681	401(58.9)	280(41.1)
		Pearson chi2(3) = 18.8609 Pr = 0.000			Pearson chi2(3) = 14.8631 Pr = 0.002		
**Injury**	No	1675	958(57.2)	717(42.8)	1471	808(54.9)	663(45.1)
**/disability**	Yes	489	281(57.5)	208(42.5)	407	232(57)	175(43.0)
		Pearson chi2(1) = 0.0113 Pr = 0.915			Pearson chi2(1) = 0.5548 Pr = 0.456		
**Area dis-**	Least disadvantaged	834	489(58.6)	345(41.4)	742	395(53.2)	347(46.8)
**advantage**	Mid-disadvantaged	772	445(57.6)	327(42.4)	675	357(52.9)	318(47.1)
	Most disadvantaged	699	372(53.2)	327(46.8)	559	336(60.1)	223(39.9)
		Pearson chi2(2) = 4.9963 Pr = 0.082			Pearson chi2(2) = 8.0405 Pr = 0.018		

Walking frequency differed by age, household type and dominant household occupation. Most frequent walking occurred among a higher proportion of those aged 55–64 years and 65 years and older, people residing in a household where there no one in the labor force, and those living in households without children.

Table 2 presents the distribution of the outcomes across each of the kernel density measures.

**Table 2:** Destination intensity by walking frequency and physical activity sufficiency.

		Walking frequency			Physical activity sufficiency		
Exposure variable	Response category	Total n(%)	3 times a week or less	At least four times a week	Total n(%)	Insufficiently active	Sufficiently active
			n(%)	n(%)		n(%)	n(%)
	Total	2305	1306 (56.7)	999 (43.3)	1976	1088(55.1)	888(44.9)
**Kernel**	Quintile 1	475	299(63.0)	176(37.1)	420	254(60.5)	166(39.5)
**Density:**	Quintile 2	488	284(58.2)	204(41.8)	427	236(55.3)	191(44.7)
**400m**	Quintile 3	420	241(57.4)	179(42.6)	366	199(54.4)	167(45.6)
	Quintile 4	462	249(53.9)	213(46.1)	384	205(53.4)	179(46.6)
	Quintile 5	460	233(50.7)	227(49.4)	379	194(51.2)	185(48.8)
		Pearson chi2(4) = 16.4032 Pr = 0.003			Pearson chi2(4) = 7.7893 Pr = 0.100		
**Kernel**	Quintile 1	489	305(62.4)	184(37.6)	434	261(60.1)	173(39.9)
**Density:**	Quintile 2	435	260(59.8)	175(40.2)	386	225(58.3)	161(41.7)
**800m**	Quintile 3	498	290(58.2)	208(41.8)	426	233(54.7)	193(45.3)
	Quintile 4	454	238(52.4)	216(47.6)	370	191(51.6)	179(48.4)
	Quintile 5	429	213(49.7)	216(50.4)	360	178(49.4)	182(50.6)
		Pearson chi2(4) = 20.6157 Pr = 0.000			Pearson chi2(4) = 12.5297 Pr = 0.014		
**Kernel**	Quintile 1	475	295(62.1)	180(37.9)	426	250(58.7)	176(41.3)
**Density:**	Quintile 2	507	310(61.1)	197(38.9)	438	265(60.5)	173(39.5)
**1200m**	Quintile 3	450	262(58.2)	188(41.8)	381	218(57.2)	163(42.8)
	Quintile 4	427	221(51.8)	206(48.2)	354	182(51.4)	172(48.6)
	Quintile 5	446	218(48.9)	228(51.1)	377	173(45.9)	204(54.1)
		Pearson chi2(4) = 25.5112 Pr = 0.000			Pearson chi2(4) = 22.9420 Pr = 0.000		

For physical activity sufficiency, the quintiles with greatest destination intensity contained a significantly higher proportion of those who were sufficiently physically active at both 800m and 1200m. At 800m, 50.6% of those in Quintile 5 were sufficiently active compared to 39.9% of those in Quintile 1. Similarly at 1200m, 54.1% of those in Quintile 5 were sufficiently active compared to 41.3% of those in Quintile 1. A higher proportion of those who were insufficiently physically active lived in areas of lowest destination intensity (60.1% of those in Quintile 1 were insufficiently active compared to 49.4% of those in Quintile 5 at 800m, and 58.7% of those in Quintile 1 were insufficiently active at 1200m, compared to 45.9% of those in Quintile 5).

As for physical activity, for each of the exposure measures a higher proportion of those walking four times a week or more lived in areas of greatest destination intensity (49.4% of those in quintile 5 walked 4/week or more compared to 37.1% in quintile 1 at 400m, 50.4% compared to 37.6% at 800m and 51.1% compared to 37.9% at 1200m). A higher proportion of those walking least frequently lived in areas of lowest destination intensity (63.0% walking 3/week or less in quintile 1 compared to 50.7% in quintile 5 at 400m, 62.4% in quintile 1 compared to 49.7% in quintile 5 at 800m, and 62.1% in quintile 1 compared to 48.9% in quintile 5 at 1200m).

### Multilevel analysis: kernel density estimation

In results for the multilevel analyses, odds ratios for quintile four and five, for each kernel distance, were similar for the physical activity and walking outcomes.

**Fig 2:**
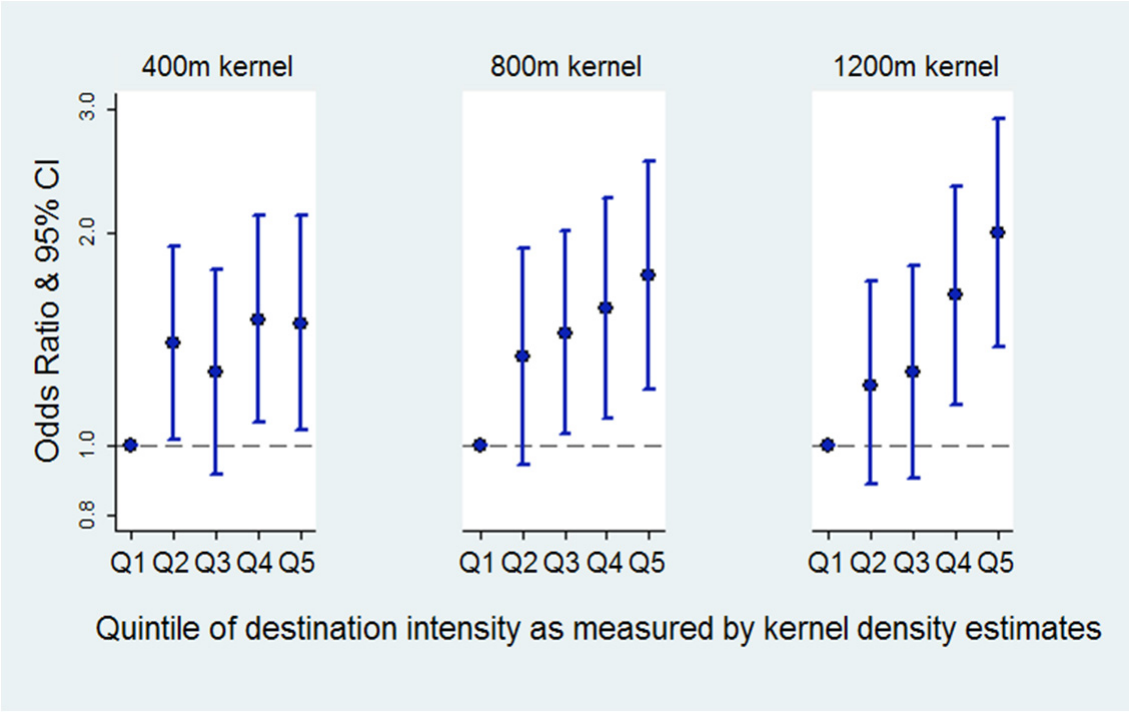
Physical activity and destination intensity: odds ratios for physical activity sufficiency compared to insufficient activity, at three kernel distances. *note: reference group for outcome: insufficient level of physical activity. **models adjusted for: age, sex, country of birth, dominant household occupation, education, disability, area level disadvantage

Associations between physical activity sufficiency and kernel density estimates are presented below (Fig 2). Significant associations between physical activity sufficiency and kernel density estimates for destination intensity were found at 400m (quintile 2 OR 1.40, 95% CI 1.02–1.91; quintile 4 OR 1.51, 95% CI 1.08–2.12; quintile 5, OR 1.49 95% CI 1.05–2.12), 800m (quintile 3 OR 1.44, 95%CI 1.04–2.01, quintile 4 OR 1.56, 95% CI 1.09–2.24; quintile 5, OR 1.74, 95% CI 1.20–2.53) and 1200m (quintile 4, OR 1.63, 95% CI 1.14–2.33; quintile 5, OR 2.00 95% CI 1.38–2.90).

**Fig 3:**
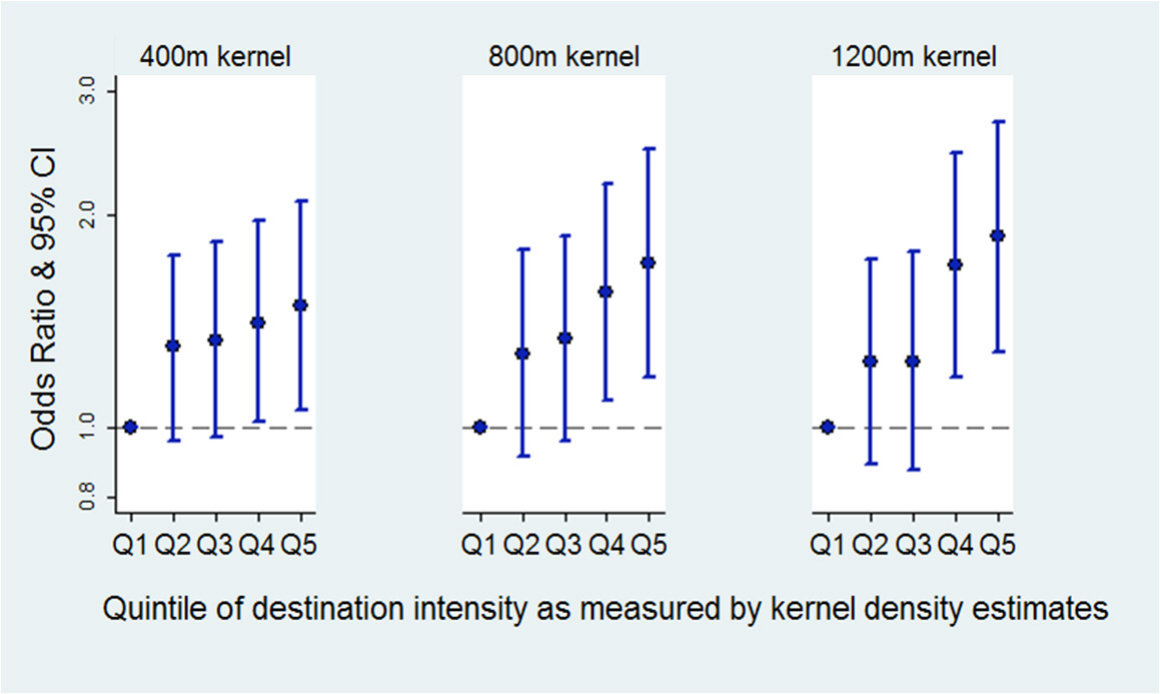
Walking and destination intensity: odds ratios for walking four times a week or more compared to walking `three times a week or less, at three kernel distances. *note: reference group for outcome: walking three times a week or less. **models adjusted for: age, sex, country of birth, dominant household occupation, education, disability, area level disadvantage

Increasing kernel density estimates for destination intensity were associated with a greater likelihood of being a more frequent walker at all kernel sizes (Fig 3). Evidence was strongest for quintile 4 and 5 relative to quintile 1 at 400m (quintile 4 OR 1.41, 95% CI 1.02–1.96; quintile 5, OR 1.49 95% CI 1.06–2.09), 800m (quintile 4 OR 1.55, 95% CI 1.09–2.21; quintile 5, OR 1.71, 95% CI 1.18–2.48) and 1200m (quintile 4, OR 1.7, 95% CI 1.18–2.45; quintile 5, OR 1.86 95% CI 1.28–2.71).

**Fig 4:**
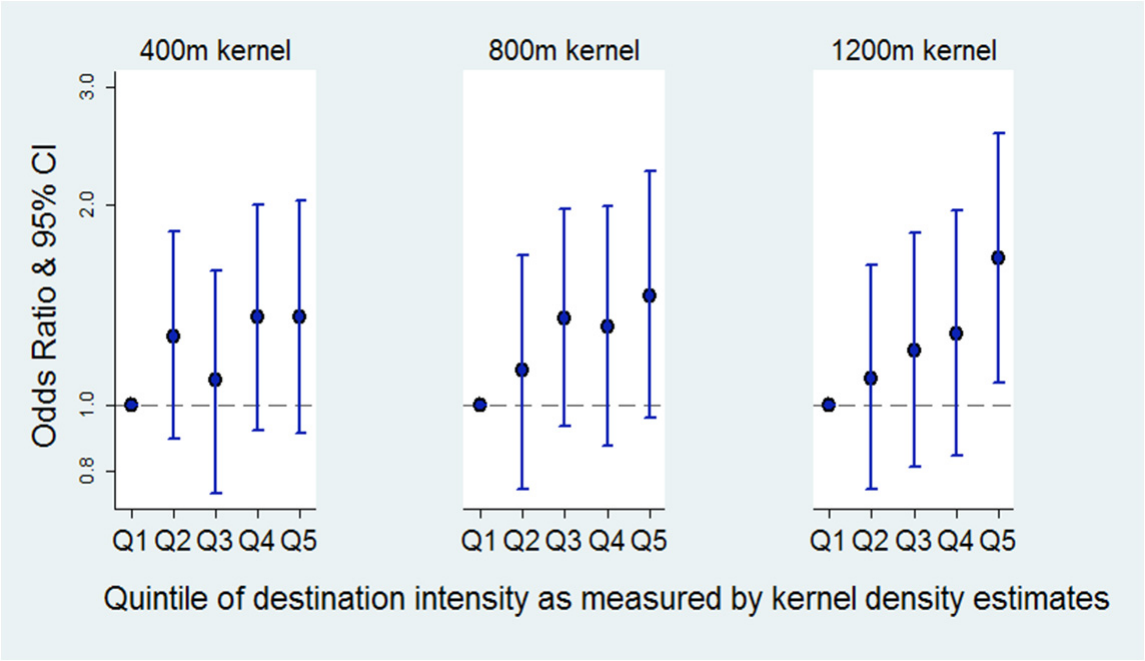
Mediation analysis models: odds ratios for physical activity sufficiency compared to insufficient activity, adjusted for walking. *note: reference group for outcome: insufficient level of physical activity. **models adjusted for: age, sex, country of birth, dominant household occupation, education, disability, area level disadvantage and walking

Mediation analysis showed that associations between destination intensity and sufficient physical activity were largely attenuated when walking was included in the models (Fig 4).

## Discussion

This study provides the strongest evidence to date of the association between destination intensity and walking and physical activity. The results show that the intensity of destinations is associated with a higher frequency of walking and physical activity sufficiency. At all kernel distances, respondents in the upper two quintiles (with the greatest destination intensity) had approximately 1.4–2.0 times the odds of being sufficiently active, and of walking at least 10 minutes, four times a week or more. The fact that walking largely attenuated the associations between destination intensity and sufficient physical activity suggests that changes to the distribution of destinations has the potential to increase physical activity, largely through walking, such that more residents are sufficiently active for health.

As there is little previous research investigating the distribution of destinations using kernel density estimation, it is difficult to place these results within an existing body of literature. Broadly, the results are consistent with studies examining the relationship between walking and the presence [18, 45], proximity [46, 47], and number of destinations [48, 49]. The results are also consistent with results of a New Zealand study in which a Neighborhood Destination Accessibility Index—a composite measure of destination proximity, diversity and intensity (number within a defined area) [17]—was found to be positively associated with accelerometer-derived, and self-reported measures of physical activity and walking [50].

The only other study that we are aware of that has used kernel density estimates in relation to a physical activity outcome found that the distribution of recreational resources was positively associated with physical activity [30]. Using a potential accessibility model (similar to KDE), Charriere and colleagues found that built environmental patterns characterized by greater accessibility to facilities and destinations (as well as high accessibility to green spaces and high density of cycle paths) was significantly associated with a higher likelihood of walking and cycling in France [51].

In making sense of the results, it is certainly plausible that greater intensity of destinations is associated with higher levels physical activity, and that this effect is mostly enacted through increased levels of walking. Cycling, which is another mode of active transport that might be used to reach destinations, is very infrequent among Australian adults [52]. In most cases, higher KDE values indicate greater clustering of destinations: having a clustered set of destinations in the neighborhood means that a range of shopping tasks/activities can be completed in a trip locally. Given the time constraints that affect most people, it is likely that many people would make a judgment that destinations dispersed around the neighborhood would take too long to access by foot. The ability to complete multiple tasks close to home provides great incentive to walk. It is also possible that areas with more clustered destinations are more congested with car traffic and have better pedestrian infrastructure, thereby increasing the viability of walking as a travel option.

It is possible that different types of destinations exert different influences on physical activity and walking for different population groups. Schools, for instance, may exert a greater effect on walking for households with young families. Cafes and community resources may encourage walking and physical activity by offering opportunities for social interaction and engagement. Unlike cars, public transport does not take travelers door-to-door (travelers need to get to and from the stop or station). The presence of public transport stops and stations may therefore encourage walking and physical activity among people accessing public transport. Given the high number of transport related destinations in the dataset, we considered the possibility that estimates of destination intensity (as measured by KDE) reflected the presence of only one or two types of destinations (such as transport destinations). We therefore conducted sensitivity analysis in which we excluded transport destinations. The pattern of results was consistent with those drawing on the full suite of destinations.

In other sensitivity analysis, we used a continuous walking measure and found that the results corroborated those obtained using the binary walking variable: increasing levels of destination intensity was associated with more minutes of walking. This was significant at quintile 5 for all distances.

It is possible that the associations observed between destination clustering and both walking and physical activity were confounded by BMI. People with lower BMIs may: 1) self-select into areas in which destinations are clustered; and 2) walk more/ be more physically active. To examine this, we conducted sensitivity analysis that adjusted for BMI. Results only changed marginally with the inclusion of BMI, with many estimates remaining unchanged. This suggests that BMI was not a confounder of the relationship between destination clustering and both walking and physical activity.

There are clear policy implications of this work. The results provide guidance for urban planners, suggesting that greater intensity of destinations may facilitate higher levels of physical activity and walking (and thereby confer health and lifestyle benefits to residents). The provision of services and infrastructure is costly, so there are substantial cost benefits if destinations can be located 800m and 1200m from most homes, rather than 400m. Furthermore, greater public health benefits are likely to arise if people walk to destinations that are further away. In light of this, evidence that more intensely distributed destinations are associated with physical activity and walking at 800m and1200m (in addition to 400m) is important, as it suggests that people may walk to services and shops that are far enough from home to offer health benefits.

### Strengths and limitations

This present analysis using KDE of destination distribution represents an important advancement in the study of the relationship between destinations and physical activity and walking. Until relatively recently neighborhood exposures were mostly based on territorially or administratively defined area units [21]. KDE offers a graded measure of exposure (destination access), avoiding the use of binary measures based on relatively arbitrary boundaries. Additionally, the use of KDE to create neighborhood areas specific to each individual optimized the specificity of exposure measures in this analysis. Furthermore, KDE methods are relatively easy to utilize, and the results improve understanding of the relationship between destinations and physical activity and walking.

The comprehensive environmental data collection methods deployed here (individual surveys, objective environmental audits by trained staff, and the use of publicly available spatial datasets) represent an important strength. The contemporaneous collection of individual and environmental data reduced the risk of bias associated with the misclassification of environmental exposures. The use of three different kernel sizes (400m, 800m and 1200m) represents another strength of this study, as it enabled the comparison of effects at different distances, thereby providing greater understanding of the complexities of walking choices and behavior.

Few previous studies have examined such a comprehensive and diverse list of destinations, particularly of both commercial and non-commercial destinations. While not exhaustive, the wide-ranging list of destinations used here is also an important strength.

Whilst novel approaches have been used here, some limitations must be acknowledged. Firstly, the results reveal associations between walking and physical activity, and destinations, however as with all cross-sectional studies, it is not possible to establish causality. Having said this, while reverse causality cannot be ruled out, it seems unlikely that high levels of walking in the neighborhood for example, leads to higher intensity of destinations or more destinations. Secondly, there is a risk of confounding due to residential self-selection. The issue of residential self-selection arises because people who are motivated to be physically active and walk rather than drive, may choose to live in an area that is suited to such behaviors [53]. This study did not collect information as to why people live in their areas. It is therefore possible that residential self-selection may overestimate associations between destination intensity and physical activity and walking. Importantly however, some studies that have adjusted for self-selection have reported an attenuation of associations, but have noted that the associations remain [53, 54]. It should also be acknowledged that while an estimate of overall walking was used in this analysis, the determinants of walking vary according to walking purpose (i.e. for transport or recreation). By not distinguishing between walking purpose, it is possible that some imprecision in, or mis-estimation of, the association between destinations and walking was introduced. Unfortunately the VicLANES survey did not enable reliable distinction between transport and recreational walking. Further, it is not known whether the reported walking occurred in each individual’s local environment or elsewhere (e.g. between a train stop and workplace). Related to this, both outcome measures were self-reported. Self- reported measures of physical activity are considered to be less sensitive than more objective measures of physical activity such as accelerometers [55]. Importantly too, it is also possible that destination intensity is correlated with other unmeasured aspects of the built environment that predict walking and physical activity. Finally, as the participants in this study were adults, the extent to which the results can be generalized to other populations such as children, the elderly and disabled, is limited.

## Conclusions

This is the first study that the authors are aware of to use kernel density estimation to assess the relationship between the distribution of a wide range of neighborhood destinations and both walking and physical activity. The results suggest that destination intensity has the potential to support residents in meeting physical activity sufficiency guidelines, with evidence that greater intensity of destinations is associated with an increased frequency of walking, as well as higher levels of physical activity sufficiency. The clear attenuation of results that occurred with the inclusion of ‘walking’ in physical activity models, suggests that the effects of destination intensity on physical activity largely operates through walking.

These findings have important policy implications, both in relation to the planning of new suburbs, and in terms of planning decisions for services and facilities in existing suburbs.

## Supporting Information

S1 Table Types and Sources of Destination Data. (DOC)
