# Supplementary material for: The Use of Kernel Density Estimation to Examine Associations between Neighborhood Destination Intensity and Walking and Physical Activity
Source: PLoS One. 2015 Sep 10;10(9):e0137402. doi: 10.1371/journal.pone.0137402 (PMC4565552; doi:10.1371/journal.pone.0137402)
Supplement: S1 Table — (DOC) [file pone.0137402.s001.doc]

**Supplementary Table S1: Types and Sources of Destination Data**

| **Data Provider** | **Data** |
| --- | --- |
| Street directories (Melway Pty Ltd) | - Childcare centers & kindergartens |
|  | - Schools |
|  | - Other places of education |
|  | - Public libraries |
|  | - Galleries, cinemas, theaters, museums |
|  | - Neighborhood houses |
|  | - Community health centers |
|  | - Community centers |
|  | - Places of worship/churches |
|  | - Maternal and child health centers |
|  | - Post offices |
|  | - Tennis courts |
|  | - Swimming pools |
| VicLANES environmental audits | - Food purchasing destinations:   - Supermarkets   - Fruit & vegetable stores   - Butchers   - Bakeries   - Ethnic food stores   - Specialty food stores   - Minor/convenience food stores   - Other food stores   - Cafes/takeaway stores |
|
|
| Metlink (Public transport operator for the Victorian State Government) | - Tram stops - Bus stops |
| PSMA (Public Service Mapping Agency) Australia Limited | - Railway lines - Railway stops - Tram/light rail lines |
